# Effect of Eccentric Calcification of an Aortic Valve on the Implant Depth of a Venus-A Prosthesis During Transcatheter Aortic Valve Replacement: A Retrospective Study

**DOI:** 10.3389/fphys.2021.718065

**Published:** 2021-08-06

**Authors:** Lanlan Li, Yang Liu, Ping Jin, Jiayou Tang, Linhe Lu, Guangyu Zhu, Chennian Xu, Yanyan Ma, Jian Yang

**Affiliations:** ^1^Department of Cardiovascular Surgery, Xijing Hospital, Air Force Medical University, Xi’an, China; ^2^School of Energy and Power Engineering, Xi’an Jiaotong University, Xi’an, China; ^3^Department of Cardiovascular Surgery, General Hospital of Northern Theatre Command, Shenyang, China

**Keywords:** transcatheter aortic valve replacement, aortic valve stenosis, eccentric calcification, Venus-A prosthesis, implant depth

## Abstract

**Object:**

Our goal was to assess the implant depth of a Venus-A prosthesis during transcatheter aortic valve replacement (TAVR) when the areas of eccentric calcification were distributed in different sections of the aortic valve.

**Methods:**

A total of 53 patients with eccentric calcification of the aortic valve who underwent TAVR with a Venus-A prosthesis from January 2018 to November 2019 were retrospectively analyzed. The patients were divided into three groups (A, B, and C) according to the location of the eccentric calcification, which was determined by preprocedural computerized tomography angiography (CTA) images. The prosthesis release process and position were evaluated by contrast aortography during TAVR, and the differences in valve implant depths were compared among the three groups. The effects of different aortic root structures and procedural strategies on prosthesis implant depth were analyzed.

**Results:**

Eleven patients had eccentric calcification in region A; 19 patients, in region B; and 23 patients, in region C. The patients with eccentric calcification in region B had a higher risk of prosthesis migration (10.5% upward and 21.1% downward), and the position of the prosthesis after TAVR in group B was the deepest among the three groups. When eccentric calcification was located in region A or C, the prosthesis was released at the standard position with more stability, and the location of the prosthesis was less deep after TAVR (region A: 4.12 ± 3.4 mm; region B: 10.2 ± 5.3 mm; region C: 8.4 ± 4.0 mm; region A vs. region B, *P* = 0.0004; region C vs. region B; and *P* = 0.0360). In addition, the left ventricular outflow tract (LVOT) (*P* = 0.0213) and aortic root angulation (*P* = 0.0263) also had a significant effect on implant depth in the aortic root structure of the patients. The prosthesis size was 28.3 ± 2.4 in the deep implant group and 26.4 ± 2.0 in the appropriate implant group (*P* = 0.0068).

**Conclusion:**

The implant depth of the Venus-A prosthesis is closely related to the distribution of eccentric calcification in the aortic valve during TAVR. Surgeons should adjust the surgical strategy according to aortic root morphology to prevent prosthesis migration.

## Introduction

Calcified aortic valve stenosis is a common valvular disease seen in older patients. In recent years, a large number of clinical studies have shown that transcatheter aortic valve replacement (TAVR) can effectively treat people at high risk for this kind of surgery (Smith et al., 2011; Himbert et al., 2012; Mack et al., 2015; Popma et al., 2019). The normal aortic valve is a symmetrical tricuspid aortic valve (TAV), however, about 2% of the population is bicuspid aortic valve (BAV) (Tchetche et al., 2019). According to the number of raphes, BAV can be divided into type0, type1, and type2 (Sievers and Schmidtke, 2007). Studies have shown that patients with BAV are more likely to cause calcified stenosis (Jilaihawi et al., 2015; Tchetche et al., 2019; Vincent et al., 2021). The distribution of aortic valve calcification is symmetrical or eccentric, and patients with eccentric distribution are difficult to operate on, which seriously increases the risk of complications after an interventional valve implant (Blanke et al., 2010; Ewe et al., 2011; Feuchtner et al., 2013; Di Martino et al., 2017). Some studies have shown that improper placement of the delivery system at the aortic root increases the complications associated with TAVR, such as paravalvular leakage (PVL), conduction block, and coronary artery occlusion, which are closely related to the depth of the prosthesis implanted into the left ventricular outflow tract (LVOT) (Delgado et al., 2010; Schultz et al., 2011; Makkar et al., 2013; Piazza et al., 2016; Wang et al., 2018), and calcification at the location of the prosthesis release, which is positively correlated with postoperative PVL (John et al., 2010), However, at present, relatively few researchers have studied the relationship between the location of aortic valve eccentric calcification and the depth of prosthesis implantation. Based on the fact that the degree and location of aortic valve calcification and the location of the release of the prosthesis affect complications associated with TAVR, this study retrospectively investigated the effect of the distribution of eccentric calcification of the aortic valve on the depth of the Venus-A valve implant after the TAVR operation in patients with eccentric calcification of the aortic valve. The results can be used to predict preoperatively the difficulty and complications of the operation.

## Materials and Methods

### Research Objects

From April 2018 to November 2019, a total of 53 patients with severe eccentric calcification of the aortic valve were selected from 128 patients with aortic valve stenosis who received a Venus-A prosthesis (Venus MedTech, Inc., Hangzhou, China) via TAVR in the department of cardiovascular surgery of the Xijing Hospital. CTA scanning and analysis of the aortic root were performed before TAVR. Eccentric calcification is defined as follows: eccentric index = (1−calcification volume of contralateral area/maximum calcification volume) × 100%. Inclusion criteria were severe eccentric calcification of the aortic valve (eccentric index > 0.6) and patients with moderate or severe calcification of the aortic valve (calcification volume > 400 mm^3^). Among the 53 patients selected, 42 were men (79.2%), and 11 were women (20.8%); the average age was 68.1 ± 7.2 years. 11 patients (20.8%) had a standard TAV; 17 patients (32.1%) had a type 0 BAV, and 25 patients (47.2%) had a type 1 BAV.

### Preoperative Computerized Tomography Angiography Protocol and Analysis

The CTA images of patients in our center were obtained using dual-source Flash CT scanners (SOMATOM Definition Flash CT scanner, Siemens, Erlangen, Germany), using retrospective electrocardiographic gating to collect the best systolic and diastolic images; the scanning range was from the aortic arch to the bottom of the heart. The settings of the equipment parameters were as follows: 2 × 32 × 0.6 mm collimation; 0.75-mm slice thickness; 0.5-mm slice interval; 100 kV tube voltage (if body mass index > 30 kg/m^2^, 120 kV tube voltage), 0.28 s/cycle frame rotation speed; 0.2–0.5 pitch; scanning direction, and head to foot. The contrast agent was injected with a three-phase protocol: first, 350 or 370 mgI/ml, 70–80 ml contrast agent at a rate of 4–5 ml/s; second, 350 or 370 mgI/ml, 20 ml contrast agent at a rate of 1.5 ml/s; and finally, 30–40 ml of normal saline at 4–5 ml/s. The CTA images were evaluated by 3mensio software (3mensio Structural Heart, Pie Medical Imaging BV, Maastricht, Netherlands). The aortic root structure was measured by the 30–45% systolic phase, and the aortic valve calcification score was calculated by the calcification volume proposed by Callister (the calcification threshold range was set at 850 HU) (Callister et al., 1998; Leber et al., 2013; Jilaihawi et al., 2014).

### Grouping Method

**FIGURE 1:**
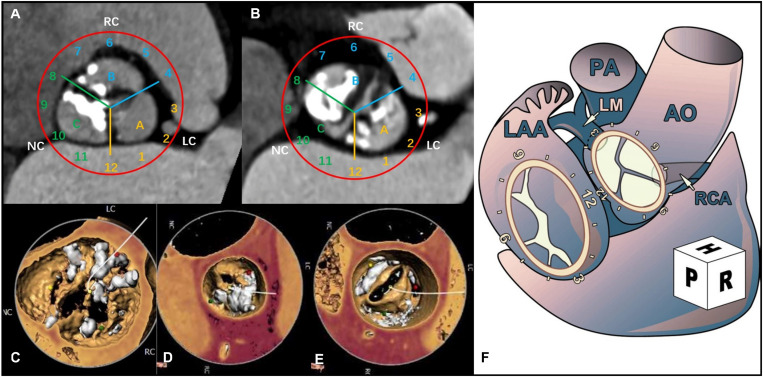
Computerized tomography angiography (CTA) shows the distribution of aortic valve calcification using a surgeon’s view clock-face model. **(A)** The CTA cross section shows the distribution of the tricuspid aortic valve (TAV) calcification. **(B)** The CTA cross section shows the distribution of the bicuspid aortic valve (BAV) calcification. **(C)** 3-Dimensional (3D) view of the CTA image shows the eccentric calcification of the aortic valve in region A. **(D)** 3D view of the CTA image shows eccentric calcification in region B. **(E)** 3D view of the CTA image shows eccentric calcification in region C. **(F)** Surgical view of the left atrium (Ruiz et al., 2011).

According to the location of the eccentric calcification, the aortic valve annulus was divided into three regions: A, B, and C. Using the surgical view, the aortic annulus was distributed according to the numbers on a clock: 12 o’clock was connected to area A2 of the anterior mitral valve; 3 o’clock was near the left atrial appendage: region A [near the left coronary artery (LCA)] was between 0 and 4 o’clock; region B [near the right coronary artery (RCA)] was between 4 and 8 o’clock; and region C was between 8 and 12 o’clock (Ruiz et al., 2011; Schultz et al., 2011; Figure 1).

**FIGURE 2:**
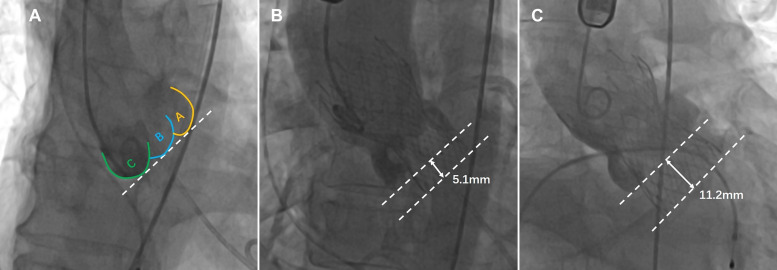
The implantation depth of the Venus-A prosthesis was measured postoperatively from fluoroscopic images (the dashed lines represent the annular plane and the Venus-A valve base plane, respectively). **(A)** Distribution of three calcified regions on fluoroscopic images; **(B)** standard implantation depth; and **(C)** implantation position that is too deep.

The distance between the Venus-A valve base plane and the annular plane was measured by an instant angiogram after implantation to determine whether the prosthesis was implanted too deeply (Piazza et al., 2016; Tang and Kaneko, 2018). The optimal depth of the Venus-A prosthesis is 4–10 mm below the aortic annulus. In this study, the depth of the prosthesis in the deep implantation group was > 10 mm, and the depth of the prosthesis was ≤ 10 mm in the non-deep implantation group (Wang et al., 2018; Figure 2).

### Statistical Analysis

SPSS 26.0 software (SPSS, Chicago, IL, United States) was used for the statistical analyses. Measurement data were expressed by numerical values; normal distribution measurement data were expressed by mean ± standard deviation ($\bar{x}\pm s$); the independent sample *t*-test was used to analyze the data between the two groups, and single factor analysis of variance was used to compare the differences between groups. There was a significant statistical difference (*P* < 0.05).

## Results

### Clinical Results

**FIGURE 3:**
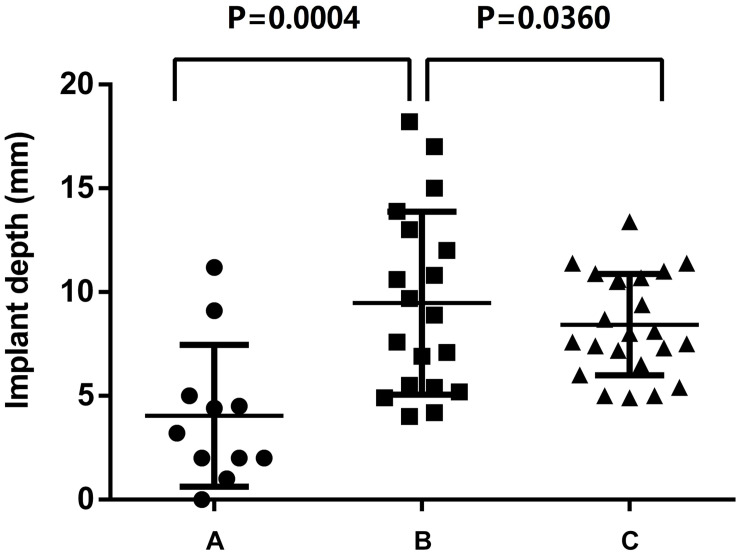
Effect of eccentric calcification of aortic valve in region **(A–C)** on implant depth of Venus-A prosthesis.

Among the 53 patients included in the study, TAV patients were 11 cases (20.8%) and BAV patients were 42 cases (79.2%), complications such as coronary artery occlusion, and conduction block occurred in two cases (3.8%). Valve-in-valve surgery was performed in four cases (7.5%) due to severe perivalvular leakage caused by the prosthesis implant. Two patients (3.8%) had prosthesis migration and re-release of the prosthesis during the operation; there were no other serious complications or surgical failures. The statistical results of the TAVR operations according to the different locations of eccentric calcification of the aortic valves were as follows (Figure 3).

Region A: In 11 patients, 2 TAV patients (18.2%), 3 type0 BAV patients (27.3%) and 6 type1 BAV patients (54.5%). The implant depth was 4.12 ± 3.4 mm; the implant was too deep in 1 case (9.1%) and not too deep in 10 cases (90.9%), of which 1 case (9.1%) had a RCA occlusion.

Region B: In 19 patients, 3 TAV patients (15.8%), 10 type0 BAV patients (52.6%) and 6 type1 BAV patients (31.6%). The implant depth was 10.2 ± 5.3 mm; the implant was too deep in 8 cases (42.1%) and not too deep in 11 cases (57.9%), of which 4 cases (21.1%) had severe perivalvular leakage and were treated with valve-in-valve surgery. The valve was transferred to the sinus of Valsalva (SOV) and withdrawn in two cases (10.5%).

Region C: In 23 patients, 6 TAV patients (26.1%), 4 type0 BAV patients (17.4%) and 13 type1 BAV patients (56.5%). The implant depth was 8.4 ± 4.0 mm; the implant was too deep in 8 cases (34.8%) and non-deep in 15 cases (65.2%), of which 1 case had left bundle branch block (4.3%).

The statistical results showed that when the calcification was located in regions A and C, the depth of the Venus-A implant was acceptable. In particular, when the area of the eccentric calcification was located in region A, the prosthesis was usually in the standard position. When the eccentric calcification was concentrated in region B (near the right coronary cusp), the risk of complications from TAVR was highest (31.6%). When the Venus-A prosthesis was implanted deeply and it was therefore easy to release the prosthesis too deeply, and the prosthesis landing location was somewhat deep, the valve-in-valve approach was needed in TAVR. When the prosthesis was located 4 mm or less under the standard annulus, the stent at the positioning point appeared to be adducted during the release process, and the contralateral stent slipped, with the result that the stent at the positioning point had to be displaced to the SOV. Regardless of whether the Venus-A prosthesis is moved up to Valsalva sinus or too deeply down, it needs to be reloaded and released during the operation.

### Preoperative Aortic Root Anatomy

In the group in which the Venus-A prosthesis was implanted too deeply, the diameter of the LVOT and the angulation of the aortic root were relatively larger (*P* < 0.05), but there was no significant difference in valve implantation depth in relation to other aortic root structures.

### Intraoperative Situation of TAVR

The intraoperative Venus-A prosthesis selection and the hemodynamic changes pre- and post-TAVR are shown in Table 1. The size of the Venus-A prosthesis in patients with a deep implant was larger than that of the size in the non-deep implant group (28.3 ± 2.4 vs. 26.4 ± 2.0; *P* = 0.0068). The peak pressure gradient of the aortic valve was significantly reduced after TAVR [(70.1 ± 35.9) mmHg vs. (6.6 ± 7.2) mmHg]. There was no significant difference in the preoperative peak pressure gradient [(59.2 ± 31.2) mmHg vs. (74.6 ± 38.8) mmHg; *P* = 0.2683] and postoperative peak pressure gradient [(5.4 ± 6.0) mmHg vs. (7.1 ± 7.7) mmHg; *P* = 0.5507] between the deep group and the non-deep group of the Venus-A recipients.

**TABLE 1:** Comparison of intraoperative conditions between deep implantation group and non-deep implantation group of the Venus-A prosthesis.

Intraoperative situation	Deep implantation group (*n* = 17)	Non-deep implantation group (*n* = 36)	Total (*n* = 53)	*P-*value
Venus-A size ($\bar{x}\pm s$)	28.3 ± 2.4	26.4 ± 2.0	26.9 ± 2.3	0.0068
Oversize ($\bar{x}\pm s$)	4.1% ± 8.4%	2.8% ± 9.6%	3.2% ± 9.3	0.6299
Preoperative peak pressure gradient (mmHg, $\bar{x}\pm s$)	59.2 ± 31.2	74.6 ± 38.8	70.1 ± 35.9	0.2683
Postoperative peak pressure gradient (mmHg, $\bar{x}\pm s$)	5.4 ± 6.0	7.1 ± 7.7	6.6 ± 7.2	0.5507

*Oversize = (Venus-A size/annular diameter−1) × 100%.*

## Discussion

Since the first successful TAVR was performed in France in 2002, the technology has been widely developed worldwide, and the indications for this operation have expanded from middle- and high-risk surgical patients to low-risk patients with aortic stenosis (Waksman et al., 2019; Coylewright et al., 2020). With the popularization of this technique, more and more patients are expected to undergo TAVR in the future. There are many approaches for TAVR, of which the femoral artery is the preferred choice. However, it is relatively difficult to adjust the position during the release of the self-expanding prosthesis during TAVR via the transfemoral route, especially for patients with severe calcified aortic stenosis. In addition, intraoperative prosthesis implantation is often too deep, resulting in severe PVL (Sherif et al., 2010), conduction block (Piazza et al., 2008), or mitral regurgitation and other adverse events (Piazza et al., 2016). Therefore, it is extremely important to study how the structure of the aortic root affects the depth of prosthesis release when making TAVR preoperative risk predictions and determining indications for the operation.

The Venus-A prosthesis, the first domestic product for TAVR produced in China, can be used effectively to treat older patients with aortic stenosis (Jilaihawi et al., 2014), this product is a self-expandable prosthesis, and the specifications are mainly determined according to the diameter of the aortic annulus. Ideally, the prosthesis is fixed to the aortic annulus by radial support force. The bottom of the prosthesis stent is covered by porcine pericardium, with a height of about 10 mm. The diameter of the narrowest part of the stent waist is 4–6 mm smaller than that of the anchor at the bottom of the stent. Because the deep position of the Venus-A prosthesis during the operation is equivalent to the smaller specification, and the anchor position is beyond the porcine pericardium, severe PVL can result (Sherif et al., 2010; Liao et al., 2017). The Venus-A system is delivered mainly from the femoral artery. Due to the long access, it is relatively difficult to control the release of the prosthesis at the aortic annulus, and the release is mainly affected by the aortic root structure. Especially when the calcification of the aortic valve is serious and uneven, there is a significant difference in the position of the prosthesis after release. At present, the Venus-A prosthesis in clinical use in China is basically a first-generation product that cannot be recycled to the sheath for readjustment during the release process. Therefore, it is particularly important for the surgeon performing TAVR to understand the structure of the landing zone before beginning the operation.

Some researchers have found that the location and severity of aortic valve calcification are independent predictors of adverse clinical outcomes (Rosenhek et al., 2000). The results of our study showed that for patients with severe calcified aortic stenosis, the impact of calcified plaque at different locations on the aortic leaflet on the implant depth of the prosthesis was different. During TAVR via the femoral approach, fluoroscopic images showed that the Venus-A prosthesis was delivered to the aortic annulus close to the great curvature of the aortic wall as it passed through the ascending aorta (except for the patients in whom the surgeon used a snare), and the landing zone of the prosthesis was located mainly in region C, that is, near the non-coronary leaflet. When the eccentric calcification was located there, it could support the release of the prosthesis and prevent the prosthesis from moving to the left ventricle. However, when the initial position of the landing zone was high, the bottom of the prosthetic stent was squeezed inward. Especially for the type 1 BAV patient with severely calcified raphe at the junction of regions B and C, the landing zone of the prosthesis will be elevated by calcification. When the prosthesis was released in this state, the bottom of the stent contacted the LVOT late, and the prosthesis was difficult to be provided timely support, which was easy to cause migration and led to deep implant. Similarly, if the calcified plaque was located in region B, and the prosthesis was not coaxial to the aortic root, the calcification could exacerbate prosthesis migration. Finally, due to severe calcified stenosis and small effective orifice area of the aortic valve, stent migration could be effectively prevented when eccentric calcification was located on the opposite side of the initial landing of the prosthesis (region A). Therefore, for these patients, the landing zone of the prosthesis should be appropriately high to ensure that the ideal depth of the prosthesis. Through analysis of images from a large number of patients having TAVR, we found that a small number of patients had a situation that was the opposite of that just described. Therefore, we analyzed statistically the anatomical structure of the aortic root and the valve specifications selected. The Venus-A prosthesis is anchored at the aortic root mainly by the annulus, the LVOT, and the calcified leaflet. Table 2 shows that when the diameter of the LVOT and the angulation of the aortic root are small, the implantation depth of the Venus-A prosthesis is mostly in the standard range. Chan et al. (2013) and Abramowitz et al. (2016) also confirmed this result. The annulus diameter and the aortic calcification volume had little effect on patients with moderate to severe aortic stenosis, which was consistent with the results of the study that selected a downsized prosthesis according to the characteristics of the supra-annulus of the patients with severe aortic stenosis (Xiong et al., 2019).

**TABLE 2:** Comparison of aortic root computerized tomography angiography (CTA) measurements between the deep implantation group and the non-deep implantation group of the Venus-A prosthesis.

Aortic root	Deep implantation group (*n* = 17)	Non-deep implantation group (*n* = 36)	Total (*n* = 53)	*P-*value
Annular diameter	27.3 ± 3.2	25.9 ± 2.9	26.5 ± 3.0	0.1325
(mm, $\bar{x}\pm s$)				
LVOT diameter	28.5 ± 3.8	26.0 ± 3.3	26.9 ± 3.9	0.0213
(mm, $\bar{x}\pm s$)				
SOV diameter	33.0 ± 5.2	34.3 ± 4.8	33.9 ± 5.0	0.3905
(mm, $\bar{x}\pm s$)				
Calcification volume	575.6 ± 272.0	797.1 ± 412.3	730.6 ± 383.7	0.1858
(850 HU, mm^3^, $\bar{x}\pm s$)				
Aortic root angulation	54.9 ± 9.3	47.7 ± 10.4	50.6 ± 10.4	0.0263
(°, $\bar{x}\pm s$)				
LCA height	13.8 ± 3.5	15.7 ± 3.9	15.0 ± 5.1	0.1186
(mm, $\bar{x}\pm s$)				
RCA height	16.6 ± 4.0	16.7 ± 3.1	16.6 ± 4.7	0.4705
(mm, $\bar{x}\pm s$)				
STJ diameter	33.5 ± 3.9	32.3 ± 5.2	32.6 ± 7.1	0.4356
(mm, $\bar{x}\pm s$)				

*All diameters measured by CTA in the table were calculated from the circumference. Aortic root angulation, angle between plane of aortic valve annulus and horizontal plane; LVOT, left ventricular outflow tract; SOV, sinus of Valsalva; LCA, left coronary artery; RCA, right coronary artery; STJ, sinotubular junction.*

We found that a larger prosthesis was used in the patients with deep implants. According to our experience, the release of the Venus-A prosthesis is divided into two stages. In the first stage, one-third of the prosthesis is released: The purpose of this process is to locate the prosthesis and ensure that it is at the standard depth. In the second stage, the prosthesis is released quickly and completely. At present, the first-generation Venus-A prosthesis, which cannot be retrieved and repositioned, is used mainly in China. Thus, the first step in the TAVR procedure is extremely important. However, when using a large prosthesis (such as 32-mm Venus-A), it is difficult at this time to control the delivery device after releasing one-third of the prosthesis: The prosthesis is released from the sheath quickly, and the operator does not have enough time to adjust the delivery system, so it is often implanted too deeply. Although the diameter of the LVOT is smaller than that of the aortic annulus, the prosthesis can be prevented from moving down properly.

## Conclusion

Older patients with aortic stenosis can be effectively treated with TAVR, and the position of the eccentric calcification on the aortic valve affects the implant depth of the Venus-A prosthesis. At the same time, the smaller LVOT diameter and the angle of the aortic root inhibit the downward movement of the prosthesis when it is released. Therefore, although the risk of prosthesis migration and PVL is high in patients with eccentric calcification of the aortic valve undergoing TAVR, the operator can predict the difficulty of TAVR by analyzing the location of the eccentric calcification before the operation. He or she can also select the appropriate prosthesis size and release position and formulate solutions to various risks that could occur during the procedure, thereby ensuring the safety and effectiveness of the procedure. In recent years, the retrievable and repositionable functions have been added to the second-generation Venus-A plus prosthesis (Liu, 2018). With the wide application of this product in the future, the safety and effectiveness of TAVR for patients with severe aortic valve calcification and eccentricity will be significantly improved.

## Limitation

Transcatheter aortic valve replacement patients selected for inclusion in this study are all from one center, and the sample size is small. The distribution area of aortic valve eccentric calcification is only divided into three parts, without considering the difference between bicuspid and TAVs. However, with the extensive development of TAVR, the study can be further refined after increasing the patient sample size. In addition, the Venus-A prostheses used in our center will be downsized for patients with severe aortic calcification according to the structure of the supra-annulus. This method can provide easier device manipulation and reduce the risk of prosthesis migration.

## Data Availability Statement

The original contributions presented in the study are included in the article/supplementary material, further inquiries can be directed to the corresponding author.

## Author Contributions

LLi conceived, analyzed, and wrote the study. YL and JY performed the project supervision and content modification. PJ and JT performed the patient diagnosis and data collection. LLu performed the statistical analysis. GZ, CX, and YM collected and measured the image data of patients. All authors contributed to the article and approved the submitted version.

## Conflict of Interest

The authors declare that the research was conducted in the absence of any commercial or financial relationships that could be construed as a potential conflict of interest.

## Publisher’s Note

All claims expressed in this article are solely those of the authors and do not necessarily represent those of their affiliated organizations, or those of the publisher, the editors and the reviewers. Any product that may be evaluated in this article, or claim that may be made by its manufacturer, is not guaranteed or endorsed by the publisher.
